# Facilitators and Barriers of Assistive Technology and Learning Environment for Children with Special Needs

**DOI:** 10.1155/2018/3705946

**Published:** 2018-12-04

**Authors:** Suchitporn Lersilp, Supawadee Putthinoi, Theeratorn Lersilp

**Affiliations:** ^1^Department of Occupational Therapy, Faculty of Associated Medical Sciences, Chiang Mai University, Thailand; ^2^Department of Special Education, Faculty of Education, Chiang Mai Rajabhat University, Thailand

## Abstract

The purpose of this research was to study the facilitators and barriers of assistive technology (AT) and the learning environment for children with special needs in special education schools in Chiang Mai, Thailand. The informants were one hundred and sixteen children with special needs, who studied in nursery to Grade 12, or with their caregivers. The instrument was a questionnaire applied by the International Classification of Functioning, Disability and Health (ICF) and examined for content validity by five specialists. The results in terms of AT showed that a majority of children with physical disability needed it for mobility and use of school buildings and those with hearing disability for communication. However, most of the children did not need to use AT for culture, recreation, or sports, while many considered it as a facilitator for education. In terms of the learning environment, most characteristics of the physical environment were facilitators for children with special needs, as were those of the social environment for all groups of such children. The results of this study were useful in providing information for AT and design of a learning environment relating to the varied characteristics of children with special needs in special education schools.

## 1. Introduction

Education is an occupation performance area that is meaningful for children, in that the school system can provide direct impact on their occupation [1]. Children with special needs require a specific education program and related services that encourage them to learn with peers in schools and participate in social activities as children do in general. Children with special needs include those with visual, hearing, physical, and intellectual disability, autism, and language and communication disorders as well as multiple disabilities. They have various needs depending on severity and context. Some children with disabilities are encouraged to study in mainstreaming schools, where they meet their needs successfully, but others do not. A number of children need more specific help and services in order to be suitable for study in special education schools.

Assistive Technology (AT) is any product, instrument, strategy, service, or practice used by people with disabilities and also the elderly. It is produced specially or availed generally to prevent, compensate, relieve, or neutralize impairment, disability, or handicap and improve the individual's autonomy and quality of life (QOL) [2]. AT users vary from children to elderly people. The purpose of AT use depends on age relating to requirement, lifestyle, and the environment in which it is used [2]. Children with special needs can use AT as a way of improving functions and encouraging learning, especially when participating in a school environment. In the school year 2015–2016, survey data of the US Department of Education indicated that a higher percentage of students aged between 3 and 21 years received special education services [3]. This high percentage showed that most of these students had learning disabilities and studied in general schools. The trend for mainstreaming and inclusive education schools is increasing in order to encourage and enable students with special needs to have the same learning environment as that of their peers without disabilities [4]. However, due to policy and socio-cultural factors, most children with special needs in Thailand enroll in special education schools, including those for the blind and with hearing, physical, and intellectual disability. Students in special education schools are provided AT from the Ministry of Education. AT items are found in schools that are related to the disabilities of their students, for example, magnifiers, Braillers, slates, and styluses in schools for the blind; hearing aids, cochlear implants, and sign language interpreters in schools for students with hearing disability; walkers, crutches, canes, wheelchairs, and Augmentative and Alternative Communication (AAC) in schools for students with physical disability; and Computer-Assisted Instruction (CAI) and educational board games in schools for students with intellectual disability [5]. Some studies reported that all types of AT items were provided to students with disabilities in special education schools. Students with visual disability were given and used more AT items than other students with disabilities, in both special education schools and universities [5, 6]. Moreover, students with visual disabilities needed AT the most, while those with hearing and physical disabilities did not need it so much [6]. Previous studies surveyed AT provision, problems, and needs, but they did not study or explore the facilitators and barriers of AT for students in the context of their learning environment. Although matching equipment with the needs and characteristics of disabled students is not always difficult, understanding the nature of their tasks or functions and environment and preferences is necessary [2]. Thus, the perspectives of students with special needs on AT as facilitators and barriers are useful for service providers when choosing, planning, and encouraging these people to reach their potential.

A learning environment refers to the diverse physical locations, contexts, and cultures in which students learn [7]. Thus, the learning environment is not only the typical classroom but also a collaboration area that can be characterized as a place for active interactions between learner and instructor or between learner and other learners [8]. Some studies found that the learning environment, especially the classroom, had a positive impact on the academic performances of the students [9, 10], and it is very important for those with special needs. A positive learning environment can facilitate the performance areas in daily life, education, play, leisure, and social participation for the students. On the other hand, a negative learning environment can obstruct their performances and skills.

International Classification of Functioning, Disability and Health (ICF) was adopted in 2001 by the World Health Organization (WHO) [11]. The ICF provides a common language to describe human functioning in people with and without disabilities. In fact, it can be a model that guides selection measures, treatment goals, and outcomes [12]. The definition of “environmental factors” in the ICF is a component that makes up the physical, social, and attitudinal environment in which people live and conduct their lives [11]. These factors include products and technology, natural environment and human-made changes to the environment, support and relationships, attitudes and services, and systems and policies. These environmental factors may act as facilitators and also barriers in meaningful activities, in which persons are able to participate [12].

The context in this study was placed on special education schools for students with disabilities, including schools for the blind, deaf and hard of hearing, and those for students having physical and intellectual disability. These schools are placed in urban areas, except for those for physical disability, which are located in suburban areas. The students are selected for admission to a school by type of disability. In addition, the schools have an evaluating team that generally comprises special education teachers, occupational therapists, and school administrators who consider minimum performances that students are expected to have, particularly regarding communication and self-care performance. Most of the students are provided AT by the rights of children with disabilities, as indicated in the Acts of the Ministry of Health. However, in some cases, students still do not receive AT; therefore, the Individual Education Plan (IEP) team plays a role in considering suitable AT for students through the rights in the Acts of the Ministry of Education. School buildings for students in need are designed in the same way as general schools and each one has three to four floors. Not all of them have elevators. However, the buildings of the school for students with physical disability do have slopes between each floor and wide walkways to facilitate wheelchairs and walking aids for the students.

In fact, AT and the learning environment are able to improve the study and participation of students with disabilities to the level of their peers. However, different types of students have varied needs. As AT and the learning environment are considered as both facilitators and barriers in schools where disabled students can participate in learning and social activities, this study is aimed at exploring these factors for providing appropriate AT and setting a suitable learning environment for these people.

## 2. Methods

This research studied the facilitators and barriers of AT and the learning environment for students with disabilities in special education schools, in Chiang Mai, Thailand. The participants were selected by stratified random sampling from all four special education schools in Chiang Mai such as those for students with physical, intellectual, hearing, and visual disability. They comprised 116 students with disabilities, including 10 kindergarten, primary, and secondary students from each school, except for only 6 kindergarten students from the school for the blind. Most of the students were boarders, but some attended day school. Students who had multiple disabilities were not included in this study.

The instrument used was a questionnaire that applied to the ICF and consisted of two parts: AT and the learning environment (Table 1). The questionnaire also was examined for content validity by five related specialists. The AT part (IOC = 0.91) included 63 items and comprised five types of AT such as that for mobility (IOC = 0.93), communication (IOC = 0.93), education (IOC = 0.94), culture, recreation, and sports (IOC = 1.00), and school buildings (IOC = 0.88). The learning environment part (IOC = 0.95) consisted of two types: physical (IOC = 0.94) and social environment (IOC = 1.00).

There were four qualifier scales in the questionnaire (F = Facilitator, B = Barrier, NO = No Opportunity, and NA = Not Applicable). An AT facilitator was defined as a convenience with positive effect on accessibility to learning and socializing activities in the school context. An AT barrier was defined as an inconvenience with negative effect on accessibility to learning and socializing activities in the school context. No Opportunity was defined as students who were aware that AT was useful for them, or they needed to use it, but did not have the opportunity to do so. Not Applicable was defined as students who did not consider AT as being necessary. The definitions of these four qualifier scales were explained to the participants individually.

An individual interview was carried out directly in the data collection process by using the questionnaire with students who had good communication skills in the Thai language. However, if the students were too young or had communication problems, the caregivers or teachers gave the interview instead. All of the kindergarten students and the adults responded to the questions in this study. Most of the primary school students (70.00%) and all of the secondary ones responded to the questions by themselves. Twenty-four adults responded to the questions on behalf of the kindergarten and 30% of the primary school students, including 8 special education teachers, 4 occupational therapists, and 12 guardians. The special education teachers and occupational therapists responded on behalf of more than one student. The conditions under which data were collected consisted of an individual interview in a quiet room. The time-span for completing the questionnaire was about 30–45 minutes. If the students or informants did not know about any type of AT, the researchers and two research assistants could give more explanation and present pictures. Then, data were analyzed by descriptive statistics for examining and summarizing the facilitators and barriers of AT and the learning environment for students with disabilities in special education schools.

## 3. Results

The students with disabilities comprised 30 with physical (25.86%), 30 with intellectual (25.86%), 30 with hearing (25.86%), and 26 with visual disability (22.42%). Most of them were female (56.90%) and 13–18 years old (41.38%). The oldest students were 18 years old and the youngest 5. In addition, two students with hearing disability had medical conditions such as hepatitis B (0.86%) and seizures (0.86%), as presented in Table 2.

The results in terms of AT showed that most of the students did not need to use it for communication, mobility, or use of school buildings, except for those with hearing and physical disability. Most of them did not need to use it for culture, recreation, or sports, and most considered AT for education as a facilitator. In terms of AT for mobility (as shown in Table 3), most of the students with physical disability perceived it as a facilitator, especially the standard wheelchair. On the other hand, many of them had no opportunity to use highly functional mobility aids such as an electric wheelchair, a scooter, or hand bicycle. In addition, many students with visual disability perceived a white cane and reflective tape as facilitators. These AT items were necessary mobility devices for students with visual disability, but it was interesting that some of them had no opportunity to use them, which were nonetheless low-tech devices that were not difficult to provide.

In terms of AT for communication (as shown in Table 4), most of the students with disabilities realized the importance of this AT as a facilitator, particularly those with hearing disability, as they probably needed it to help them. It was interesting that many students with visual disability considered AT for communication as a facilitator, even though they had no obvious communication barriers. When considering detail, this study found that most students with visual disability perceived AT for communication as a facilitator for written communication.

In terms of AT for education (as shown in Table 5), most of the students perceived this AT as not necessary for this purpose. However, most of those with intellectual disability indicated that concentration training software was a facilitator for them in education, while students with physical disability needed AT items for participating in classroom educational activities such as pen and notebook, concave table, and touch screen computer.

In terms of AT for recreation (as shown in Table 6), many of the students perceived the importance of AT for recreation as a facilitator. At the same time, many of them had no opportunity to use it, except for those with visual disability. This finding showed that although this AT was in the educational context, the students perceived it as necessary.

In terms of AT for school buildings (as shown in Table 7), most of the students with physical disability reported that this AT was a facilitator, particularly the guide post for all types of these students. However, even though a low number of students with visual disability reported on AT for school buildings, they indicated that the permanent and movable slope was a barrier for them. This information is useful, as it shows that not all AT is a facilitator for all students with disabilities. It depends on what their disabilities are. That is to say, some AT is able to be a facilitator for some types of students with disabilities but not for others.

There are two parts in terms of the learning environment, including the physical and social environment. Results for the physical environment showed that it was mostly a facilitator for students with physical disability, except for the colored bands on school building stairs, braille keypads, and elevators, which were not applicable. The physical environment for students with intellectual disability was mostly a facilitator, except for obstacles in school buildings, such as the barriers of slopes and steep stairs. In addition, the students had no opportunity to use the colored bands on stairs or elevators in school buildings. The physical environment for students with hearing disability was mostly a facilitator, except for the barriers of slopes and colored bands on school building stairs. In addition, results indicated that stairs, slopes, colored bands on stairs, classroom doors, steps in front of classroom doors and position of handles in a classroom, use of the doorknob in the classroom, weight of a chair, and use of an elevator in a school building were not applicable. The physical environment for students with visual disability was mostly a facilitator, except for the elevator, which the students had no opportunity to use (as shown in Table 8).

In terms of the social environment, all four groups of students with special needs perceived it as a facilitator (as shown in Table 9). However, except for those with physical disability, the students perceived this AT as a barrier in relation to the school context. Furthermore, some students with intellectual and hearing disability reported that help from assistant teachers was not necessary for them.

## 4. Discussion and Conclusion

This study is aimed at exploring the facilitators and barriers of AT and the learning environment for students with special needs in special education schools in Chiang Mai, Thailand, by using a questionnaire that applied to the ICF. The results of this study found not all students indicating AT for mobility as a barrier. Most of these AT items were perceived as facilitators by the students with physical disability, while no students with hearing disability saw them as such. On the other hand, most AT items for communication were perceived as a facilitator for students with hearing disability. These findings indicated that not all AT is necessary for all students with disabilities, but necessity depends on their type of disability [12]. Students with physical disability have the problem of general mobility, while those with visual disability frequently need AT to help them move and gain access to where they need to communicate and participate in social activities [13, 14]. In fact, students with communication difficulty, such as those with hearing and intellectual disability as well as cerebral palsy, also need AT for accessing and participating in their daily activities [15]. However, some AT items might be a facilitator and barrier simultaneously. For instance, while AT for school buildings might be a facilitator to enable access for students with physical disability, it might be a barrier in the case of those with visual disability. Therefore, awareness of the students' perspectives should be considered, and they should be able to participate in the process of AT design and provision if possible [16]. These findings relate to previous studies [5, 6], which also indicated that most AT items were perceived as facilitators by students with disabilities, but their necessity depends on the students' disability. For example, the slopes in school buildings are a facilitator for students with physical disability, while being a barrier for students with visual disability, although slopes are designed universally for all people. When considering the design of slopes in school buildings for blind students, this study found that most slopes had no rail on either side. For this reason, some blind students might fall down when walking along the edge of a slope. In other words, whether AT items are facilitators or not depends on how they are able to compensate for disability, and this is why awareness of the students' perspectives should be considered in the process of AT design and provision. Besides AT being provided for all students with disabilities, there must be awareness of quality and design. Moreover, the findings showed that students reported NO for most AT items or they did not have the opportunity to use high-tech AT. Students with physical disability did not have the opportunity to use high-tech AT related to mobility and education such as an electric wheelchair or adapted keyboard. Students with intellectual disability did not have the opportunity to use high-tech AT such as a mobile phone, which related to communication. Students with hearing disability did not have the opportunity to use high-tech AT such as an FM system, which also related to communication. Lastly, students with visual disability did not have the opportunity to use high-tech AT such as a Thermoform Brailon Duplicator, which related to communication as well. This information reflected that some needs for high-tech AT items were not reached by children with disabilities, although they were addressed in the rights of students with disabilities under ministerial regulations. This was due to a problem with management policy and national socio-economic status. Most high-tech AT items are expensive and so many steps have to be taken before receiving them. In addition, the AT items addressed in ministerial regulations are provided only for students who studied in mainstreaming schools and are in the Individual Education Plan (IEP). On the other hand, most students with disabilities are placed in special education schools in Thailand and some have no IEP. These problems are barriers against AT provision and interesting points for further study.

This study found that the physical environment, in terms of the learning environment, is a facilitator for all types of students with disabilities. This is because this study researched in special education schools that were designed for specific disabilities and suitable for limiting each type of disability. These schools are based on a universal design concept in order to encourage, particularly students with physical disability, to be able to access classrooms, because the way buildings are constructed has direct impact on student participation [17]. In addition, these findings indicated that the physical environment is as important as the social one. In fact, besides the physical environment, the lack of understanding and positive perspectives in society leads to barriers against improving the occupational performance of students with special needs [18]. When these students have a good relationship with their teachers, peers, other students, and other school staff, they will be motivated to improve their learning and increase their opportunity to participate in school activities [19, 20]. The findings in terms of the learning environment indicated that students and adults perceived both the physical and social environment as facilitators in special education schools by responding to the questionnaire. It is of interest that as some of the students in the physical environment need facilitators, others see them as barriers, and special education schools do not need the same building construction plans. Instead, they should construct with a barrier-free design for each type of disability. Furthermore, the social environment, including relationships with teachers, peers, other students, and other school staff, is important for all of the students. In providing information to build understanding and a positive attitude in school societies, facilitators encourage children with disabilities to participate in school and social activities in the same way as people do in general.

Previous researches surveyed the AT items that students with disabilities needed, used, and had problems using. This study provides more findings in terms of student perspectives on AT and the learning environment as facilitators or barriers. Moreover, this study reflected findings on the opportunities for students with disabilities to receive high-tech AT in provincial areas of a developing country. In addition, this study is useful for related service providers such as educators, school therapists, and administrators in that it examines the individual needs of each student and presents the AT that will meet them. These people have to be aware of a safe design for AT together with environmental evaluation and encouragement in understanding students with disabilities, in order to relate with people and build relationships in the school context. The limitation of this study was the specific area of Chiang Mai province. Further research should expand to wider areas and study mainstreaming schools that include various types of students with disabilities in the same school. Furthermore, the roles of school-based occupational therapists should cover devices and environmental adaptation, and studies on their effectiveness would be interesting for students with disabilities. In addition, further studies might research other aspects and their correlation with, for example, physical environment and mobility, social environment and communication, or learning environment and education.

## Figures and Tables

**Table 1:** Applying ICF to the questionnaire on facilitators and barriers of assistive technology and the learning environment.

ICF domains in environmental factors	The questionnaire
*Chapter 1: products and technology*	*Part 1 assistive technology*
Products or substance for personal consumption (e110)	Not included
Products and technology for personal use in daily living (e115)	Applied in (i) Assistive technology for mobility (ii) Assistive technology for communication
Products and technology for personal indoor and outdoor mobility and transportation (e120)	Applied in assistive technology for mobility
Products and technology for communication (e125)	Applied in assistive technology for communication
Products and technology for education (e130)	Applied in assistive technology for education
Products and technology for employment (e135)	Not included
Products and technology for culture, recreation, and sport (e140)	Applied in assistive technology for culture, recreation, and sports
Products and technology for the practice of religion and spirituality (e145)	Not included
Design, construction and building products, and technology of buildings for public use (e150)	Applied in assistive technology for buildings
Design, construction and building products, and technology of buildings for private use (e155)	Applied in assistive technology for buildings
Products and technology for land development (e160)	Not included
Assets (e165)	Not included

*Chapter 2: natural environment and human-made changes to the environment*	*Part 2 the learning environment*
Physical geography (e210)	Not included
Population (e215)	Applied in the physical environment
Flora and fauna (e220)	Not included
Climate (e225)	Applied in the physical environment
Natural events (e230)	Not included
Human-caused events (e235)	Not included
Light (e240)	Applied in the physical environment
Time-related changes (e245)	Not included
Sound (e250)	Not included
Vibration (e255)	Not included
Air quality (e260)	Not included

*Chapter 3: support and relationships*	
Immediate family	Not included
Extended family	Not included
Friends	Applied in the social environment
Acquaintances, peers, colleagues, neighbors, and community members	Applied in the social environment
People in positions of authority	Applied in the social environment
People in subordinate positions	Not included
Personal care providers and personal assistants	Applied in the social environment
Strangers	Not included
Domesticated animals	Not included
Health professionals	Not included
Health-related professionals	Not included

*Chapter 4: attitudes*	Not included

*Chapter 5: services, systems, and policies*	Not included

**Table 2:** Characteristics of children with disabilities (*n* = 116).

Characteristics	Numbers (percentage)				Total
	PD	ID	HD	VD	
*Gender*					
Male	9	20	13	8	50
Female	21	10	17	18	66
*Age (years old)*					
6 and below	2	10	8	4	24
7–9	12	4	6	2	24
10–12	5	5	4	6	20
13–18	11	11	12	14	48
*Class level*					
Nursery	10	10	10	6	36
G1–G6	10	10	10	10	40
G7–G12	10	10	10	10	40
*Medical condition*					
No	30	30	28	26	114
Yes	0	0	2	0	2
(i) Hepatitis B	0	0	1	0	1
(ii) Seizures	0	0	1	0	1

VD = visual disability; HD = hearing disability; PD = physical disability; ID = intellectual disability.

**Table 3:** Facilitators and barriers of AT for mobility.

Items	PD (*n* = 30)				ID (*n* = 30)				HD (*n* = 30)				VD (*n* = 26)			
	B	F	NO	NA	B	F	NO	NA	B	F	NO	NA	B	F	NO	NA
White cane	/	/	/	30	/	/	/	30	/	/	/	30	/	13	3	10
Reflective tape	/	/	/	30	/	1	/	29	/	/	/	30	/	11	6	9
Walker	/	5	/	25	/	/	/	30	/	/	/	30	/	/	/	26
Crutch	/	2	/	28	/	/	/	30	/	/	/	30	/	/	/	26
Cane	/	2	/	28	/	/	/	30	/	/	/	30	/	/	/	26
Standard wheelchair	/	23	/	7	/	/	/	30	/	/	/	30	/	/	/	26
Electric wheelchair	/	1	18	11	/	/	/	30	/	/	/	30	/	/	/	26
Hand bicycle	/	2	12	16	/	/	/	30	/	/	/	30	/	/	/	26
Scooter	/	/	15	15	/	/	/	30	/	/	/	30	/	/	/	26

**Table 4:** Facilitators and barriers of AT for communication.

Items	PD				ID				HD				VD			
	B	F	NO	NA	B	F	NO	NA	B	F	NO	NA	B	F	NO	NA
Hearing aids (behind ears)	/	/	/	30	/	/	/	30	/	27	1	2	/	/	/	26
Hearing aids (in-ears)	/	/	/	30	/	/	/	30	/	25	3	2	/	/	/	26
Cochlea implant	/	/	/	30	/	/	/	30	/	18	12	/	/	/	/	26
FM system	/	/	/	30	/	/	/	30	/	16	14	/	/	/	/	26
Mobile phone	/	6	6	18	/	7	7	16	/	12	5	13	/	12	/	14
Binoculars	/	/	3	27	/	1	/	29	/	2	1	27	/	6	3	17
Slate and stylus	/	/	/	30	/	1	/	29	/	2	/	28	/	22	3	1
CCTV	/	/	/	30	/	2	/	28	/	4	/	26	/	/	/	26
Brailler	/	/	/	30	/	/	/	30	/	1	/	29	/	23	1	2
Thermoform Brailon duplicator	/	/	/	30	/	1	/	29	/	/	/	30	/	12	7	7
Braille notebook computer	/	/	/	30	/	/	/	30	/	/	/	30	/	20	/	6
Optical character recognition	/	/	/	30	/	1	/	29	/	/	/	30	/	3	6	17
Book reader software	/	2	/	28	/	1	/	29	/	/	/	30	/	16	1	9
Tone bar	/	/	/	30	/	1	/	29	/	1	3	26	/	/	1	25
Listen and speech training device	/	2	/	28	/	4	1	25	/	17	12	1	/	/	/	26
Sign language interpreter	/	/	/	30	/	/	/	30	/	30	/	/	/	/	/	26
Remote control	/	4	7	19	/	1	/	29	/	11	3	16	/	/	/	26

**Table 5:** Facilitators and barriers of AT for education.

Items	PD				ID				HD				VD			
	B	F	NO	NA	B	F	NO	NA	B	F	NO	NA	B	F	NO	NA
Adjustable chair	/	2	9	19	/	/	/	30	/	1	1	28	/	/	/	26
Electric desk	/	/	16	14	/	/	/	30	/	1	1	28	/	/	/	26
Concave table	/	19	4	7	/	1	/	29	/	/	1	29	/	/	/	26
Adapted keyboard	/	/	18	12	/	/	/	30	/	/	1	29	/	/	/	26
Keyboard controller	/	/	8	22	/	/	/	30	/	/	/	30	/	/	/	26
Prism glasses	/	/	/	30	/	1	/	29	/	2	/	28	/	/	/	26
Book holder	/	4	14	12	/	5	/	25	/	2	1	27	/	/	/	26
Writing and typewriting device	/	/	6	24	/	/	/	30	/	2	/	28	/	/	/	26
Book opening device	/	/	6	24	/	/	/	30	/	/	/	30	/	/	/	26
Word recognition software	/	/	2	28	/	1	/	29	/	2	1	27	/	/	/	26
Touch screen	/	19	2	9	/	13	2	15	/	5	1	24	/	/	/	26
Word prediction software	/	2	4	24	/	4	1	25	/	5	2	23	/	/	/	26
Concentration training software	/	10	4	16	/	25	/	5	/	8	1	21	/	/	/	26
Pen and notebook	/	27	/	3	/	19	10	1	/	16	/	14	/	/	/	26

**Table 6:** Facilitators and barriers of AT for recreation.

Items	PD				ID				HD				VD			
	B	F	NO	NA	B	F	NO	NA	B	F	NO	NA	B	F	NO	NA
Sport wheelchair	/	16	/	14	/	/	/	30	/	/	2	28	/	/	/	26
Adapted sport devices	/	14	4	12	/	2	/	28	/	/	1	29	/	/	/	26
Adapted musical instruments	/	5	13	12	/	9	2	19	/	2	4	24	/	6	/	20
Adapted educational games	/	18	4	8	/	27	/	3	/	14	11	5	/	16	/	10
Web camera	/	1	3	26	/	6	4	20	/	13	1	16	/	1	/	25

**Table 7:** Facilitators and barriers of AT for school buildings.

Items	PD				ID				HD				VD			
	B	F	NO	NA	B	F	NO	NA	B	F	NO	NA	B	F	NO	NA
Permanent slope	/	28	/	2	/	4	/	26	/	1	/	29	1	/	/	25
Movable slope	/	18	8	4	/	3	/	27	/	/	/	30	1	/	/	25
Automatic door	/	18	10	2	/	3	/	27	/	1	9	20	/	/	/	26
Adapted handle	/	22	6	2	/	/	1	29	/	3	/	27	/	/	/	26
Guide post	/	29	/	1	/	29	/	1	/	22	/	8	/	26	/	/

**Table 8:** Facilitators and barriers of the physical environment.

Items	PD				ID				HD				VD			
	B	F	NO	NA	B	F	NO	NA	B	F	NO	NA	B	F	NO	NA
*Buildings*																
Floor texture around the building	3	27	/	/	10	20	/	/	10	13	/	7	/	26	/	/
Steps around the building	/	30	/	/	12	16	/	2	/	11	/	19	2	24	/	/
Barriers around the building	3	20	7	/	12	9	/	9	12	5	2	11	7	19	/	/
Rail around the building	/	30	/	/	/	20	3	7	1	23	/	6	/	13	11	2
Slope into the building	/	30	/	/	/	13	5	12	12	1	2	15	/	26	/	/
Rail of slope	/	30	/	/	/	15	3	12	1	21	/	8	/	26	/	/
Barriers on the slope	/	21	/	9	15	3	2	10	12	5	3	10	1	19	6	/
Rail along the steps	/	18	2	10	/	28	/	2	2	21	/	7	/	26	/	/
Floor texture of steps	/	22	2	6	10	20	/	/	11	12	2	5	/	26	/	/
Height of each step	3	13	2	12	16	14	/	/	10	13	/	7	1	25	/	/
Width of each step	3	19	2	6	14	16	/	/	/	21	/	9	/	26	/	/
Colored tape on each step	/	6	1	23	/	6	21	3	10	9	1	10	/	16	/	10

*Classroom*																
Door sill	/	29	/	1	13	15	/	2	/	10	4	16	6	20	/	/
Door step	/	30	/	/	16	12	/	2	/	9	3	18	6	20	/	/
Floor texture at the entrance	/	30	/	/	/	17	/	13	1	22	/	7	/	26	/	/
Position of door handle	3	25	2	/	2	26	/	2	2	11	/	17	/	26	/	/
Width of door	/	30	/	/	/	30	/	/	1	21	/	8	/	26	/	/
Floor texture	/	30	/	/	/	30	/	/	1	23	/	6	/	26	/	/
Object arrangement	/	30	/	/	/	30	/	/	1	25	/	4	/	26	/	/
Number of students	/	30	/	/	12	16	2	/	13	15	/	2	/	26	/	/
Temperature	/	30	/	/	/	30	/	/	1	27	/	2	/	26	/	/
Lights	/	30	/	/	/	30	/	/	/	30	/	/	1	24	/	1
Position of switches	4	16	10	/	/	30	/	/	/	27	/	3	1	18	7	/

*Elevator*																
Width of door	/	28	2	/	/	1	26	3	1	8	3	18	/	/	26	/
Height of bottoms	1	27	2	/	/	1	26	3	1	6	2	21	/	/	26	/
Braille bottoms	/	13	2	15	/	1	26	3	2	4	3	21	/	/	26	/
Rail in elevator	/	28	2	/	/	1	26	3	/	11	2	17	/	/	26	/
Floor texture	/	28	2	/	/	1	26	3	/	11	2	17	/	/	26	/
Voice notification	/	27	3	/	/	1	26	3	/	1	26	3	/	/	26	/

*Desk and chair*																
Height of desk	/	30	/	/	13	17	/	/	/	16	/	14	/	26	/	/
Width of desk	/	30	/	/	13	17	/	/	/	25	/	5	1	25	/	/
Texture of desk	/	30	/	/	10	17	/	3	1	15	/	14	/	26	/	/
Weight of desk	/	28	/	2	13	14	/	2	1	16	/	13	6	20	/	/
Height of chair	/	30	/	/	13	17	/	/	1	16	/	13	/	26	/	/
Width of chair	/	30	/	/	10	20	/	/	1	15	/	14	/	26	/	/
Texture of chair	/	30	/	/	10	17	/	3	1	15	/	14	/	26	/	/
Weight of chair	/	28	/	2	13	17	/	/	2	13	/	15	6	20	/	/

**Table 9:** Facilitators and barriers of the social environment.

Items	PD				ID				HD				VD			
	B	F	NO	NA	B	F	NO	NA	B	F	NO	NA	B	F	NO	NA
*Relationship with teachers*																
Help from classroom teachers	/	30	/	/	/	30	/	/	/	30	/	/	/	26	/	/
Help from other teachers in the school	/	30	/	/	/	30	/	/	/	30	/	/	/	26	/	/
Help from assistant teachers	/	30	/	/	/	21	/	9	1	26	/	3	/	26	/	/

*Relationship with peers*																
Interpersonal activities	/	30	/	/	1	29	/	/	1	29	/	/	1	25	/	/
Help each other	/	30	/	/	/	30	/	/	/	30	/	/	1	25	/	/

*Relationship with other students in school*																
Interpersonal activities	/	30	/	/	/	30	/	/	/	30	/	/	1	25	/	/
Help each other	/	30	/	/	/	30	/	/	/	30	/	/	1	25	/	/

*Relationship with other school staff members*																
Help from other school staff members	/	30	/	/	/	30	/	/	/	29	/	1	/	26	/	/

## Data Availability

The data used to support the findings of this study are available from the corresponding author upon request.
